# Systematic Review Examining the Behavior Change Techniques in Medication Adherence Intervention Studies Among People With Type 2 Diabetes

**DOI:** 10.1093/abm/kaae001

**Published:** 2024-02-09

**Authors:** Vivien Teo, John Weinman, Kai Zhen Yap

**Affiliations:** Institute of Pharmaceutical Sciences, King’s College London (KCL), London, UK; Department of Pharmacy, National University of Singapore (NUS), Singapore; Institute of Pharmaceutical Sciences, King’s College London (KCL), London, UK; Department of Pharmacy, National University of Singapore (NUS), Singapore

**Keywords:** Medication adherence, Diabetes, Systematic review, Behavior change technique

## Abstract

**Background:**

Although previous systematic reviews have studied medication adherence interventions among people with Type 2 diabetes (PwT2D), no intervention has been found to improve medication adherence consistently. Furthermore, inconsistent and poor reporting of intervention description has made understanding, replication, and evaluation of intervention challenging.

**Purpose:**

We aimed to identify the behavior change techniques (BCTs) and characteristics of successful medication adherence interventions among PwT2D.

**Methods:**

A systematic search was conducted on Medline, Embase, CINAHL, PsycINFO, Cochrane Central Register of Controlled Trials, Web of Science, and Scopus. Studies were included if they were randomized controlled trials with BCT-codable interventions designed to influence adherence to anti-diabetic medication for PwT2D aged 18 years old and above and have medication adherence measure as an outcome.

**Results:**

Fifty-five studies were included. Successful interventions tend to target medication adherence only, involve pharmacists as the interventionist, contain “Credible source” (BCT 9.1), “Instruction on how to perform the behaviour” (BCT 4.1), “Social support (practical)” (BCT 3.2), “Action planning” (BCT 1.4), and/ or “Information about health consequences” (BCT 5.1). Very few interventions described its context, used theory, examined adherence outcomes during the follow-up period after an intervention has ended, or were tailored to address specific barriers of medication adherence.

**Conclusion:**

We identified specific BCTs and characteristics that are commonly reported in successful medication adherence interventions, which can facilitate the development of future interventions. Our review highlighted the need to consider and clearly describe different dimensions of context, theory, fidelity, and tailoring in an intervention.

## Teaser

Interventions targeting medication adherence only, involving pharmacists and containing specific behaviour change techniques seemed successful in improving medication adherence among people with type 2 diabetes

## Introduction

Diabetes is a growing public health concern worldwide with substantial healthcare impact. In 2017, 6.28% of the world’s population had Type 2 diabetes mellitus (T2DM). This equalled to 6,058 individuals per 100,000 and was projected to increase to 7,079 individuals per 100,000 by 2030 [1]. T2DM is a progressive chronic metabolic condition that results in micro and macrovascular complications, morbidity, and mortality [2]. People with T2DM (PwT2D) have 2–2.5 times increased risk of having a heart attack, heart failure, or stroke [3].

T2DM management is complex. The use of anti-diabetic oral medication and/or injections is the mainstay treatment. However, medication therapy may be intrusive, inconvenient, and confusing due to complicated dosing regimen. Concurrently, PwT2D need to adopt other self-care behaviors, such as exercise and diet. Additionally, they are twice as likely to suffer from depression [4, 5], which in turn negatively affects their medication adherence [6, 7].

Although the importance of medication adherence in PwT2D is well documented, adherence varies widely between 36% and 93% [8, 9]. Medication nonadherence is associated with poor health outcomes [10] and increased healthcare costs [11]. For example, each point increment in nonadherence on the Morisky Medication Adherence Scale was associated with a 0.21% increase in hemoglobin A1c (HbA1c), and more than 20% increase in hospitalization [12].

Existing systematic reviews on medication adherence interventions in PwT2D reported limited and inconclusive effectiveness [13, 14]. No intervention could be identified to improve medication adherence consistently [13, 14]. Furthermore, inconsistent and poor intervention reporting made its understanding and replication challenging, presenting a need to use standardized language such as the Behavior Change Techniques Taxonomy version 1 (BCTTv1) to describe interventions [15]. BCTT is an extensive taxonomy containing 93 behavior change techniques (BCT) [15]. A BCT is an observable, replicable, and irreducible active ingredient of an intervention that regulates the target behavior [15]. Each BCT has its own label and definitions. This promotes clear evaluation, replication, and reporting of an intervention [15].

An earlier review by Upsher et al. in PwT2D focused on BCTs in psychological interventions only for improving HbA1c, which may be affected by many variables other than medication adherence [16]. No review to date has studied medication adherence interventions using the BCTT among PwT2D and their effect on medication adherence outcome specifically. Therefore, we aim to identify the BCTs and characteristics of successful medication adherence interventions among PwT2D.

## Methodology

This review followed The Preferred Reporting Items for Systematic Reviews and Meta-analysis (PRISMA) statement [17].

### Searches

Medline, Embase, CINAHL, PsycINFO, Cochrane Central Register of Controlled Trials, Web of Science, and Scopus were systematically searched on March 14, 2022 to retrieve papers from January 2018 onwards. This publication limit was set following Upsher et al.’s previous search period [16]. The full search strategy was displayed in Supplementary Material 1. While the term “adherence” may be perceived by some as not reflecting person-centered approach sufficiently [18, 19], we chose to use the term “adherence,” because one may also propose that “adherence” reflects PwT2D’s act of will to follow treatment mutually agreed upon by PwT2D and healthcare providers, acknowledges PwT2D participation in decision-making and is an outcome of patient-centered care [20, 21]. Besides, the World Health Organization’s definition of adherence in 2003 is widely used in the studies relevant to our review and the term “adherence” was used in the Organisation to Economic Co-operation and Development recent report in 2018 [10].

### Study Selection

Studies were included if they were randomized controlled trials (RCTs) with parallel design and BCTT-codable intervention in non-inpatient settings (e.g., hospital outpatient, primary care). An intervention was considered BCTT-codable if it contains BCT directly applied to our target behavior (adherence to anti-diabetic medication), population (PwT2D aged 18 years old and above), and provides specific detail required according to the BCTTv1 definition and training. For example, “Problem Solving” (BCT 1.2) was only coded if the description contains both parts of the BCT definition, namely analyzing factors influencing the behavior and generating strategies to overcome barriers or increase facilitators. This BCT would not be coded if barrier identification was described without the solutions. Medication adherence measure and/or its related construct was a required study outcome in accessible full-text, peer-reviewed papers in English language journals.

Studies were excluded if they were gray studies, dissertation, and conference proceedings and lack specific medication adherence results for PwT2D in studies with different clinical groups (in other words, medication adherence results for different clinical groups, including PwT2D were pooled and presented collectively and medication adherence results for PwT2D specifically could not be identified). If a study consisted of different variation of medication and non-medication-related interventions and the medication adherence results were not specific to those receiving medication adherence intervention, the study was also excluded.

Screening was undertaken by two reviewers independently (V.T. as the first reviewer, N.D./S.Y./Y.K. as the second reviewer). Both reviewers screened the study title and abstract after removing duplicates using Endnote 20. If conclusion on their eligibility could not be drawn, their full articles were screened. If discrepancies could not be resolved between the two reviewers, a third reviewer (K.Z.Y./J.W.) was involved. Corresponding authors were contacted via email if further clarification was required.

### Data Extraction

Study and intervention details on the study design, country, setting, and context followed the number of target behaviors, types of interventionists, mode, dose, theory, fidelity, tailoring, and BCTs were extracted in August—December 2022. The Template for Intervention Description and Replication (TIDieR) guideline was used [22].

Intervention dose was defined by its duration, frequency, and amount, which referred to the time period, number of contact, and length of each contact respectively [23]. In light of the benefits of using theory in different stages of an intervention [24, 25] and the UK Medical Research Council (MRC)’s recommendation on program theory/logic model, our review assessed if studies explicitly applied theory in explaining reasons for nonadherence, developing and evaluating intervention, and presented a program theory/logic model.

The TIDieR guideline elaborates tailoring of an intervention by looking at whether an intervention was personalized, titrated, or adapted with description on the what, why, when, and how [22]. Our review examined whether an intervention reported any form of tailoring, described details on how it was tailored, and if it was tailored to individuals’ specific medication adherence barriers.

Similarly, the TiDiER guideline describes fidelity as the degree to which an intervention occurred as intended and explained the how and by whom if intervention adherence or fidelity was planned [22]. Therefore, we reviewed whether a study planned any fidelity procedures for different parts of an intervention such as its training, delivery, and receipt and reported the extent to which an intervention was implemented and received as intended.

A data extraction Excel form was pilot tested on six studies by the first and second reviewers, before the first reviewer completed data extraction for all studies.

### Operational Definition of a Successful Intervention: Classification Algorithm

**Fig. 1.**
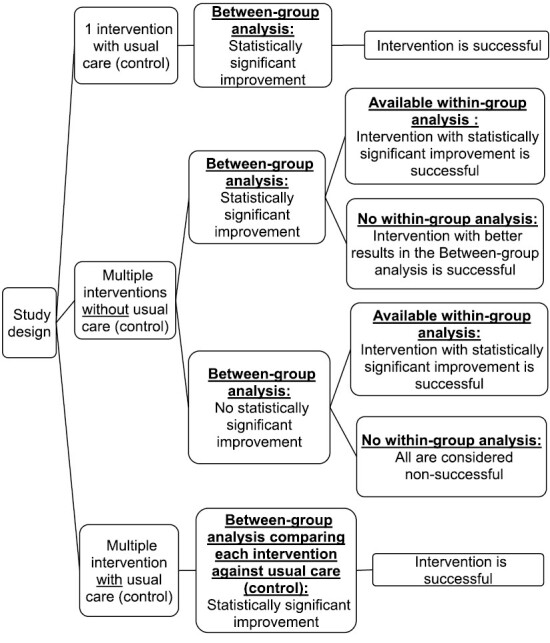
Successful intervention classification.

An intervention was considered successful if there was statistically significant improvement in one of the medication adherence measures and/or related constructs, as denoted by a *p*-value <.05 or 95% confidence interval (CI) in the between-group analysis (where calculated). In studies with multiple interventions without a control arm, a statistically significant difference in the between-group analysis may imply different permutations. For example, all interventions could be successful or nonsuccessful with significant difference in the degree of success. Hence, if within-group analysis results were available, they were also examined to aid our classification of successful interventions (Fig. 1).

### BCT Coding

Intervention description from published studies, Supplementary Materials, and protocol were reviewed to code the BCTs. BCTs in the control arm and incorporated for the conduct of the research itself, such as reward for study participation were not coded. BCT coding was first piloted on five interventions by the first and second reviewers, who undertook the BCTTv1 training [26], before both reviewers coded all interventions independently with multiple interim discussions. Any irreconcilable difference was resolved through further discussion with a third reviewer. A codebook (Supplementary Material 2) was developed to describe additional strategies, which reviewers used to code certain BCTs in medication adherence. BCT coding showed good inter-rater reliability as the mean percent agreement between the first and second reviewers was 96.9 (range 91.4%–100%) and the Gwet’s AC1 was 0.92, *p* < .001 (95% CI = 0.85, 0.98) using STATA 17 [27].

### Medication Adherence Outcomes

The type and number of specific medication adherence measures were extracted. They were categorized if their statistically significant results were clearly specific to all or only certain measurement timepoints. In our review, adherence outcomes during the follow-up period after an intervention had ended were defined as “extended post-intervention data” and were also collected to see how long an intervention effect continued.

### Risk of Bias Assessment

The Cochrane risk of bias tool for randomized trials (RoB 2) [28] was used to assess if the RCTs have low, high, or some concerns on the risk of bias. The first and second reviewers assessed the RoB for three studies independently, reconciled any disagreements before the first reviewer completed the RoB for the remaining studies.

## Results

**Fig. 2.**
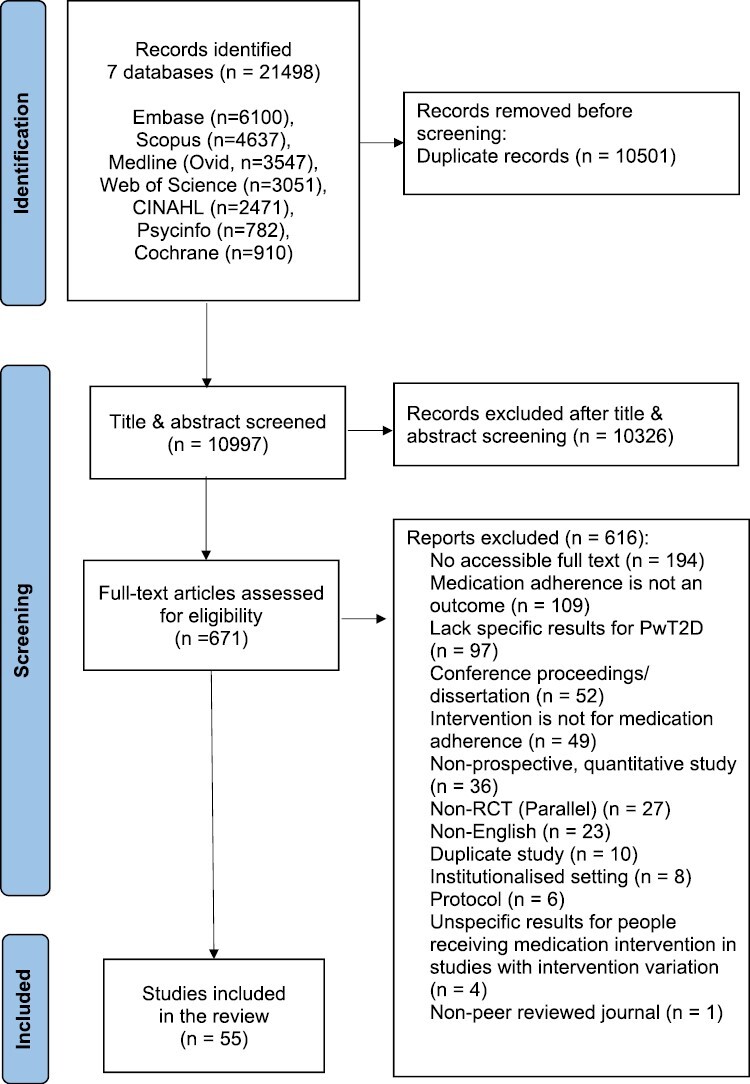
PRISMA flow chart.

In total, 10,997 title and abstracts were screened, 671 full texts were assessed, and 55 studies were included in our review (Fig. 2). Characteristics of all the studies included in this review were summarized in Supplementary Material 3. Six included studies specified suboptimal medication adherence level as an inclusion criterion [29–34]. The heterogeneous nature and the lack of information in the studies prevented further synthesis and description of the participants’ sociodemographic characteristics.

### Study Characteristics

Forty-two studies compared an intervention with usual care. The studies consisted of 30–1,022 participants [35,36]. Most studies were from the USA (*n* = 9), India (*n* = 7), and Iran (*n* = 6). One study was conducted in two countries [36]. Most studies took place at the hospital outpatient (*n* = 23) and primary health setting (*n* = 16). Four studies were performed across two settings, for example, at hospital outpatient and participants’ home [37]. About half of all the studies briefly described (50.9%) and did not describe (49.1%) the context of their interventions. Among the 28 studies that briefly described the context, 82.1% of them described only 1 dimension of the context. The most frequently described dimension was healthcare system (*n* = 24), followed by society and culture (*n* = 4).

### Intervention

There were 67 interventions in the 55 studies included. Thirty-six interventions were considered successful based on the algorithm in Fig. 1.

Of the 39 interventions that targeted multiple behaviors including medication adherence, 46.2% were successful. On the other hand, 64.3% of the interventions solely targeting medication adherence were successful.

Forty-seven interventions involved a human interventionist, 16 interventions did not report their interventionist, and 4 interventions were solely digital. Pharmacist was the most common interventionist (*n* = 29), followed by nurse (*n* = 7). Notably, 65.5% of the interventions involving a pharmacist were successful.

Most interventions (*n* = 23) were in-person individual sessions, and of these 52.2% were successful. Less than 10 interventions were conducted via text messaging, mobile app, in-person group sessions, and phone, respectively. Nine interventions did not specify their mode of delivery. Fifty-nine interventions had variable frequency of exposure depending on participants’ needs and intervention schedule, and of these 52.5% were successful. Only 25 interventions reported the length of contact for at least 1 mode in their intervention.

### Theory, Fidelity, and Tailoring

Forty-six interventions did not explicitly report the use of a theory. Most interventions that were explicit on using theory incorporated it in their development only (Table 1). No intervention featured a program theory/logic model.

**Table 1:** Theory, Fidelity, and Tailoring in Interventions

Parameter	Number of interventions	Number of successful interventions	Percentage of successful interventions with this parameter
Theory			
Not reported on using theory	46	25	54.3
Explicit on using theory	21	11	52.4
The stage where theory was used			
Explaining reasons only	3	2	66.7
Developing intervention only	13	6	46.2
Evaluating intervention only	1	0	0.0
Explaining reasons and developing intervention	3	2	66.7
Explaining reasons, developing, and evaluating intervention	1	1	100.0
Planned fidelity^a^ procedures			
Not reported	42	22	52.4
Reported	25	14	56.0
Types of planned fidelity procedures			
Training only	19	12	63.2
Delivery only	2	0	0.0
Training + Delivery	2	1	50.0
Recipient only	1	1	100.0
Delivery + Receipt	1	0	0.0
Tailoring^b^			
Not reported if tailored	20	11	55.0
Tailored	47	25	53.2
Number of tailoring types			
One	42	21	50.0
Multiple	5	4	80.0
Common types of tailoring			
Personalized review	33	17	51.5
Progress/outcome dependent	6	2	33.3
Customized pillbox/medicine packaging/ filling	5	4	80.0
Operational	5	3	60.0

^a^Fidelity is the degree to which an intervention occurred as intended.

^b^Tailoring refers to the personalization, titration, or adaptation of an intervention.

Twenty-five interventions reported planned fidelity procedures and 56.0% of them were successful. Most of the planned fidelity procedure involved training only (Table 1). Only one study reported the fidelity assessment results [38].

Thirteen interventions were explicit on using theory and reported planned fidelity procedures. Seven of these interventions were successful.

Forty-seven interventions reported tailoring in one way or another, where studies mentioned “personalised action plan” [39] and “tailored counselling” [40]. There were also operational forms of tailoring, such as arranging call frequency or text based on participants’ preference [41, 42]. Most interventions had one type of tailoring only. Of the interventions with multiple types of tailoring, 80% were successful (Table 1). Only four interventions explicitly analyzed individuals’ barriers to medication adherence and were tailored to the barriers identified [43–46]. Only two of these four tailored interventions were successful [44, 46].

### Individual BCTs

Twenty-eight individual BCTs were coded. This was equivalent to 30.1% of the BCTs in the taxonomy. Individual BCTs in all interventions are shown in Supplementary Material 4.

“Credible source” (BCT 9.1) was most frequently coded in all interventions and 58.3% of the interventions containing this BCT were successful. Most interventions with “Instruction on how to perform the behaviour” (BCT 4.1) and “Social support (practical)” (BCT 3.2) were successful too. BCTs present in more than 10 interventions with at least 57% of these interventions being successful were “Credible source,” “Instruction on how to perform the behaviour,” “Social support (practical),” “Action planning” (BCT 1.4), and “Information about health consequences” (BCT 5.1) (Table 2, more details in Supplementary Material 5). No apparent trend on the combination of individual BCTs was identified among the successful interventions.

**Table 2:** Common Individual BCTs in Interventions

Common individual BCT	Number of interventions	Number of successful interventions containing this BCT	Percentage of successful interventions with this BCT
9.1. Credible source	36	21	58.3
3.1. Social support (unspecified)	33	17	51.5
4.1. Instruction on how to perform the behavior	29	19	65.5
3.2. Social support (practical)	19	13	68.4
1.2 Problem solving	18	10	55.6
1.4 Action planning	14	8	57.1
5.1. Information about health consequences	14	8	57.1
6.1. Demonstration of the behavior	12	5	41.7
8.1. Behavioral practice/rehearsal	12	5	41.7
1.1 Goal setting (behavior)	10	3	30.0

The mean number of BCTs per intervention was 4.02, with a range of 1–12. Higher number of BCTs did not necessarily lead to successful interventions, as none of the interventions with 8–9 BCTs were successful, while 85.7% and 80% of the interventions containing 5 and 7 BCTs respectively were successful (Supplementary Material 6).

### Medication Adherence Measures

There were 33 specific types of medication adherence measure used in the 55 studies (more details in Supplementary Material 7). The most common measure was the Morisky Medication Adherence Scale (MMAS-8) (*n* = 12), followed by pill count (*n* = 7), and MMAS-4 (*n* = 5).

Medication adherence results clearly specific to the reported timepoint were found in 51 interventions, and of these 58.8% were successful. Sixteen interventions had unclear reporting whether their adherence results were specific to all or certain timepoints when they were measured. For example, medication adherence outcomes were taken at 1, 3, 6, and 12 months, but it was ambiguous if statistically significant improvement took place at all or certain timepoints only [47]. Of the interventions with such unclear results, 37.5% were successful.

Table 3 provides a summary of intervention duration in relation to medication adherence outcomes measured at the end of the intervention and extended post-intervention data. Some interventions measured medication adherence outcomes at multiple extended post-intervention follow-up timepoints, for example, Poonprapai et al. measured medication adherence 3 and 6 months after the intervention has concluded [48]. Most interventions were conducted over 6 months (*n* = 18), where medication adherence outcomes were collected at the end of the intervention without extended post-intervention data. Among the 13 interventions conducted over 3 months, 12 had medication adherence outcomes at the end of the intervention, 7 had timepoint-specific successful results, and 2 had successful 6-month extended post-intervention data at clearly specific timepoints.

**Table 3:** Summary of Intervention Duration and Medication Adherence Outcomes Measured

Intervtn duration	Number of Intervtn	Interventn with medication adherence outcomes at the end of an intervtn			Interventn with extended post-intervention data			
		Number of intervtn with outcomes at this timepoint	Number of intervtn with successful outcomes at this timepoint	Percentage of intervtn with successful results clearly specific to this timepoint^a^	Measurement timepoint from the end of an interventn	Number of intervtn with outcomes at this timepoint	Number of intervtn with successful outcomes at this timepoint	Percentage of intervtn with successful results clearly specific to this timepoint^a^
1 session only	8	0	0	0	1–2 months	4	4	75.0
					3 months	5	2	20.0
					6 and 12 months	1	1	0
2–10 weeks	6	4	2	50.0	1.5 and 9 months	1	0	0
					3 months	3	3	100.0
3 months	13	12	7	58.3	3 months	2	1	50.0
					6 months	2	2	100.0
					Around 2 years	1	0	0
14–16 weeks	3	3	2	66.7	6 months	1	0	0
6 months	18	18	8	38.9	0	0	0	0
32–36 weeks	4	3	2	66.7	3 months	1	0	0
12 months	12	11	6	27.3	1–5 months	1	0	0
					3 months	1	0	0
Not specified	3	Non-applicable						

Extended post-intervention data: adherence outcomes measured during the follow-up period after an intervention has ended.

^a^Number of interventions with outcomes at this timepoint as the denominator. *Intervtn* intervention.

### Risk of Bias

Thirty-four studies appeared to have an overall high risk of bias, while the remaining studies may have some concerns. Most studies had high risk of bias in Domain 4 Measurement of outcome (40.0%), followed by Domain 2 Deviation from intended interventions (25.5%), and Domain 5 Selection of the reported results (21.8%) (Supplementary Material 8). Among the 36 successful interventions, 50% had some concerns, while 50% had high risk of bias. Among the 31 non-successful interventions, 67.7% of them seemed to have high risk of bias (67.7%), while there might be some concerns with the remaining 32.3%.

## Discussion

Our review found that a higher number of successful interventions targeted medication adherence only, involved pharmacists as the most common interventionist, contained “Credible source” (BCT 9.1), “Instruction on how to perform the behaviour” (BCT 4.1), “Social support (practical)” (BCT 3.2), “Action planning” (BCT 1.4), and “Information about health consequences” (BCT 5.1). Very few interventions described context in detail, incorporated theory, examined extended post-intervention data, or were tailored to specific barriers identified. Poor reporting of studies was noted.

Less interventions targeting multiple behaviors were successful, as compared with those targeting medication adherence alone (46.2% vs 64.3%). Although other studies recommended changing multiple behaviors concurrently instead of one behavior at a time [49] since successful change in one behavior can encourage successful change in other behaviors [50], this was not observed in our review. Other intervention studies may be targeting a range of behaviors in multiple stakeholders at once, instead of targeting behaviors among patients only [51]. Besides, PwT2D may prioritize other self-care behaviors over medication adherence. Other self-care behaviors, such as diet, may be driven by factors different from those that drive medication adherence. Furthermore, different people may not engage in a behavior for different reasons. Hence, interventions targeting multiple behaviors may not address specific drivers underlying each behavior for an individual, resulting in lower success rates.

Although context is multi-dimensional in nature, most studies described single dimension relating to the health system, without other dimensions, such as the digital dimension on telephone usage which would be relevant to a telephone-based intervention [52]. The National Institute for Health and Care Research elucidated that understanding context is crucial in hypothesizing the effects, barriers, facilitators, implementation, adaptation, transferability, and scaling of an intervention [53]. The description of context provided by the studies in our review may be insufficient. This raises a question on the context detail that should minimally and ideally be given to attain the benefits of understanding the context. Our review found much room for improvement in describing context adequately in medication adherence intervention research.

Interventionists have a bearing on the medication adherence outcomes [54]. This explained why “Credible Source” (BCT 9.1) was commonly found in successful interventions, and it is noteworthy that 65.5% of the interventions involving pharmacists were successful. This was consistent with a meta-analysis that found pharmacists producing the largest effect sizes in delivering medication adherence interventions among people with hypertension [55]. This may be because pharmacists were seen as a credible source of information to patients [54, 55].

Among the 67 interventions, 21 of them mentioned explicit use of a theory. Benefits on the explicit use of theory in interventions have been established in existing literature [24, 25, 56]. Theory provides a logical strategy for developing interventions, evaluating their effectiveness [24], and guiding the selection of BCTs [25, 57]. Nonetheless, many interventions did not make explicit reference to theory, reflecting a general lack of using theory to inform, guide, and evaluate an intervention.

Our review concurred with other reviews that planned fidelity procedures and their reporting were lacking [16, 54, 58]. Only one type of planned fidelity procedure was found in our review, that is, training of interventionists. Studies described training with varying level of details. Training may be briefly described as “a trained pharmacist” [52] or detailed with the tools prepared for the interventionists [40]. Also, limited fidelity reporting made it challenging to determine whether the BCTs were delivered and received as planned or whether the BCTs were truly not effective in changing medication adherence behavior. Limited reporting of planned fidelity procedures explained similar percentage of successful interventions observed in interventions that reported and did not report planned fidelity procedures in our review (56.0% vs 52.4%).

Although 70.1% of the interventions reported tailoring in one way or another, the description on mechanisms of tailoring was brief or unclear. The word “tailored” or “personalised” was used without information about participant assessment and subsequent guidelines for tailoring [22]. Another review also found that, though studies reported tailoring, there was a lack of specific details on how tailoring was done and this hindered replication of the intervention [59]. Interventions could be minimally tailored, resulting in negligible benefits from tailoring, and hence similar percentage of successful interventions seen in interventions with and without tailoring (53.2% vs 55.0%).

To be more effective in overcoming barriers for medication adherence, Allemann et al. proposed that tailored interventions should match their target determinants to intervention types [60]. A systematic review described that 13% of the studies examined reasons for medication nonadherence and tailored interventions to these [59]. Only 6% of our interventions explicitly analyzed individuals’ barriers to medication adherence and were then tailored to the barriers identified. Hence, it was nearly impossible to determine if a BCT is irrelevant or ineffective in addressing a participant’s specific adherence barrier. Thus, our review supports the recommendation by Allemann et al. that adherence interventions should target specific barriers for each individual [60]. This emphasizes the need for researchers to conduct a detailed behavioral diagnosis before designing their interventions using frameworks, such as the Capability, Opportunity, Motivation, Behavior (COM-B) model and to match the BCTs to the factors identified at an individual level [58].

Certain BCTs were not found in our review. They may irrelevant or under-explored in medication adherence interventions. For example, “Behavioural Contract” (BCT 1.8) and “Commitment” (BCT 1.9) were absent in our review, but may improve medication adherence, as successful behavior change is unlikely without serious commitment [58]. Similarly, “Identity” (BCT 13) which was not noted in any intervention may be a lost opportunity. Identity is a potentially strong driver for medication adherence, as medication is an unwanted reminder of being ill and a threat to people’s sense of identity [61, 62]. Our review revealed the need to explore rarely used but potentially relevant BCTs in medication adherence interventions.

“Action planning” (BCT 1.4) and “Instruction on how to perform the behaviour” (BCT 4.1) that were present in proportionally more successful interventions in our review were also associated with significantly reduced HbA1c in another meta-analysis [16]. “Action planning” is understandably beneficial as behavior change theories proposed that action planning is the mediator between intention to change and the actual behavior change [63]. “Instruction on how to perform the behaviour” helps PwT2D to achieve the five rights required for proper medication intake: right patient, right medication, right time, right dose, and right route [64]. Hence, “Action Planning” and “Instruction on how to perform the behaviour” may be prioritized in medication adherence intervention for PwT2D.

“Information about health consequence” (BCT 5.1) and “Social support (practical)” (BCT 3.2) were also identified in more successful interventions. “Information about health consequence” was unsurprising as Leventhal’s Common-Sense Model of Self-regulation explained how consequences define people’s mental representation of current/ future health threat and treatment in managing their illness [65]. “Information about health consequence” was also frequently coded in effective interventions for illness beliefs and medication adherence in other reviews [54, 66]. Social support has strong impact on medication adherence in PwT2D [67], possibly through its overall beneficial effect (direct-effect model) or stress-protecting effect (buffering model) [68]. Hence, our review found “Information about health consequence” and “Social support (practical)” potentially helpful for medication adherence in PwT2D.

The common BCTs found in the successful interventions of our review using the BCTTv1 could be easily mapped to the closest BCTs in the BCT ontology (BCTO) that was newly released during our review (Supplementary Material 9) [69]. This review signposts the BCTs in the BCTO that future intervention studies may want to focus on.

Most studies included participants regardless of their adherence level at baseline and did not target participants who were nonadherent only. Hence, studies may not show a significant effect of an intervention that was also received by adherent participants.

More than 50% of the 3-month interventions were successful at the end of the intervention and 6 months post-intervention. Only four interventions longer than 3 months collected extended post-intervention data, and none showed statistically significant improvement. This may be because following up with patients are time and resource intensive [70], especially for long interventions. This also indicates a potential need for booster intervention sessions to maintain change in adherence as people may have different barriers to adherence requiring different interventions over time.

We have some reservations on the overall quality of the studies included. Participants’ reported outcome measure was commonly used in medication adherence studies and patients themselves were the outcome assessors. Participants were not blinded and may be influenced by their knowledge of the intervention received. Unfortunately, there was a lack of information in the studies included to rule out this possibility. Study reporting is important to ensure enough information is provided to allow evaluation of the study quality. Insufficient or unclear information leads to some concerns or high risk of bias based on the RoB2 tool. While it is difficult to do blinding for behavioral interventions, researchers may consider and explain steps to avoid participants’ reported outcomes from being influenced by their knowledge of intervention by blinding them to the group assignment and details of different arms. For example, Friedberg et al. provided a general study description without implying which arm was hypothesized to be better. They gave tailored counseling to one arm, and non-tailored counseling to another arm to control for the attention given by counseling [71].

### Strengths and Limitations

To our knowledge, this is the first systematic review, which has attempted to identify the BCTs in medication adherence interventions for PwT2D. This review included only RCTs, thus ensuring that only data from studies that provided similar level of evidence was pooled. As the geographical setting did not form part of the selection criteria of studies, our review findings were applicable to wider populations in Asian, Western, high, and lower-income countries. At least two reviewers were involved with high inter-rater reliability shown, indicating consistency in BCT coding and increasing confidence in our review. Our review also assessed the context, target behavior, theory, tailoring, and fidelity of the interventions in greater depth by highlighting the specific dimensions of each parameter present or absent in the studies. Detailed reporting of medication adherence outcomes at different timepoints further enabled the investigation of intervention effects over time.

An intervention was considered successful or not in our review based on its statistical significance. We acknowledged that statistical significance does not equate to clinically significance and it would be ideal to use clinically relevant effect, such as HbA1c as the ground for determining the success of an intervention. However, HbA1c may be affected by many variables (e.g., diet, physical activity), other than medication adherence, making it difficult to ascertain if the changes in HbA1c are due to medication adherence intervention or other factors. Therefore, to tease out the effect of an intervention on medication adherence specifically, we used the statistical significance of the adherence outcomes as the surrogate marker of its effectiveness.

A meta-analysis could not be performed due to the wide diversity in the study design, interventions, and medication adherence measures of the studies included. Other systematic reviews on medication adherence interventions have faced the same limitation [13, 66]. Therefore, descriptive analysis was performed by assessing different aspects of the study design and intervention in greater depth.

BCT coding depends on reporting quality. Similar to other reviews [16, 51, 54], our review found that intervention reporting was inadequate. A BCT may be present, but not identified due to insufficient details of the intervention. Although additional information was requested from study authors, no replies were obtained on these requests.

## Future Direction and Conclusion

The suboptimal quality of some RCTs highlighted a need for better design and/or reporting of RCTs for interventions aimed at improving adherence. Transparent and comprehensive description of the BCTs and intervention characteristics are warranted to provide clarity for future studies. Interventionists are recommended to specify BCTs as they develop an intervention to improve the reporting, understanding, evaluation, and fidelity of delivering the active ingredients due to the certainty around the BCTT. Interventions containing BCTs were significantly more effective than interventions that did not [72]. We hope that this review on specific BCTs and characteristics of successful medication adherence interventions can facilitate the development of future interventions.

## Supplementary Material

kaae001_suppl_Supplementary_Materials_1

## Transparency statements

1. *Study registration*. Study registration involves declaring the study design, variables, and treatment conditions in an independent, institutional repository (e.g., http://clinicaltrials.gov, https://osf.io/, https://www.socialscienceregistry.org/). A study is considered pre-registered if it is registered in a repository prior to the study began. This study was not formally registered. 2. *Analytic plan pre-registration*. Pre-registration of an analytic plan involves specification of the planned sequence of analyses or the statistical model that will be reported. Reporting the planned outcome variables alone does not qualify as analytic plan pre-registration. The analysis plan was not formally pre-registered. 3. *Data availability*: Indicate whether the data underlying the presented analyses will be made available in a third-party archive/repository. If data is available in an archive, indicate if this is fully available to the public or has protected access (available for limited audience, with clearly defined requirements, and steps to obtain data). If data is not in a public archive, describe any steps needed to access the data. De-identified data from this study are not available in a public archive. De-identified data from this study will be made available (as allowable according to institutional IRB standards) by emailing the corresponding author. 4. *Analytic code availability*. Analytic code refers to the programming code used to conduct the analyses in the statistical software. There is no analytic code associated with this study. 5. *Materials availability*. Materials include any stimuli used in this study, intervention protocols, and survey instruments and items. Materials used to conduct the study are not publicly available.
