# Supplementary material for: Systematic Review Examining the Behavior Change Techniques in Medication Adherence Intervention Studies Among People With Type 2 Diabetes
Source: Ann Behav Med. 2024 Feb 9;58(4):229–41. doi: 10.1093/abm/kaae001 (PMC10928844; doi:10.1093/abm/kaae001)
Supplement: kaae001_suppl_Supplementary_Materials_1 [file kaae001_suppl_supplementary_materials_1.docx]

Electronic supplementary materials are available at Annals of Behavioural Medicine online.

# Electronic Supplementary Material (ESM) 1: Search strategy

The following search strategy was used for Medline and was adapted for the other databases. Search terms are free text terms, unless otherwise stated. MESH: Medical subject heading (Medline medical index term); exp: exploded MeSH; *: truncation that searches for root word with any endings. Adj: adjacent

**Diabetes**

1. MESH: exp Diabetes Mellitus/dt [Drug Therapy]
2. Diabet*.ab,ti.
3. 1 or 2

**Medication adherence**

1. MeSH: exp Medication Adherence/
2. MeSH: exp treatment refusal/
3. MeSH: exp patient dropouts/
4. MeSH: exp Self Administration/
5. MeSH: exp Health Knowledge, Attitudes, Practice/
6. 4 or 5 or 6 or 7 or 8
7. (drug* or medication* or medicine* or therap* or treatment* or regimen* or prescription*).ab,ti.
8. (adher* or nonadheren* or non-adheren* or complian* or noncomplian* or non-complian* or persist* or concordan* or refus* or empower* or belief* or believe*).ab,ti.
9. (10) adj3 (11)
10. 9 or 10 or 11 or 12

**Combining both search concepts**

1. 3 and 13
2. Apply publication limit to 14 above in the following manner:

- For Part 1A: Systematic Review of quantitative intervention studies: From 2018 onwards
- For Part 1B: Synthesis of qualitative studies: From 2014 onwards

# ESM 2: BCT Codebook

All 93 BCTs were referred to during coding. Only BCTs requiring further clarification and have accompanying strategy that guided reviewers in the coding are listed below. Generally, if the intervention is clearly on medication adherence, individual BCT is coded even if the extracted description excerpt does not spell out medication adherence explicitly.

| Definition from BCTTv1 taxonomy | Additional coding strategy/ notes |
| --- | --- |
| **1.1 Goal setting (outcome)**  Set or agree on a goal defined in terms of a positive outcome of wanted behaviour | Coded if an intervention is clearly for medication adherence, though the extracted intervention description excerpt does not spell out medication adherence explicitly |
| **4.1 Instruction on how to perform a behaviour**  Advise/ agree on how to perform the behaviour (includes “skills training).  Note: When the person attends classes such as exercise or cookery, code BCT 4.1 Instruction on how to perform the behaviour, 8.1 Behaviour practice/ rehearsal and 6.1 Demonstration of the behaviour | Coded if the intervention involves talking about medication use (e.g., types, doses, side effects) |
| **5.1 Information about health consequences**  Provide information (e.g., written, verbal, visual) about health consequences of performing the behaviour | Coded if the excerpt is on complications of diabetes |
| **8.1 Behavioural practice/ rehearsal**  Prompt practice or rehearsal of the performance of the behaviour one or more times in a context or at a time when the performance may not be necessary, in order to increase habit and skill. | Coded for teach-back method |
| **11.3 Conserving mental resources**  Advise on ways of minimising demands on mental resources to facilitate behaviour change | Coded if the intervention involves less complex medication use, for example in terms of the device used (pen versus syringes), combination medication |

# ESM 3: Summary of study characteristics

The studies were arranged based on the presence of theory, followed by the presence of tailoring

| **Study** | **Comparator** | **Intervention description** | **What (Theory)** | **Who** | **How (Mode)** | **When/ How much** | **Tailor** | **How well (fidelity process planned)** |
| --- | --- | --- | --- | --- | --- | --- | --- | --- |
| Ranjbaran et al, 2022 | Usual care | Education | Y | NR | FF, phone & msg; Indv & Grp | Variable over unclear duration | Y | NR |
| Farmer et al, 2021 | Usual care | Digital msg | Y | NR | msg | 3-4 times a week over 12 months | Y | NR |
| Dang et al, 2020 | Usual care | Good Literacy to Enhance Response | Y | Pharmacist | FF, phone; Indv | Variable over 3-month | Y | Y |
| Benson et al, 2019 | Usual care | diEtitiaNs Helping pAtieNts CarE for Diabetes | Y | Dietitian | Phone; Indv | Variable over 1-year | Y | Y |
| Nelson et al, 2021 | Usual care | Education/ Encouragement | Y | NR | Msg | Variable over 12-month | Y | Y |
| Trevisan et al, 2020 | Usual care | Implementation intention | Y | NR | FF, phone; Indv | 4 visits and 2 calls over 15-week | Y | NR |
| Varming et al, 2019 | Usual care | Empowerment and motivation | Y | Nurse | FF & phone; Indv | 4 visits and 1 call over 3.5-month | Y | Y |
| Gautier et al, 2021 | Usual care | Personalized test messages | Y | Pharmacist | Msg | Daily over 3-month | Y | Y |
| Najafpour et al, 2021 | Usual care | Education | Y | Doctor, psychiatrist, nutritionist | FF, msg, phone; Indv, Grp | Variable over 3-month | Y | NR |
| Razmara et al, 2018 | Usual care | Family-Based Follow-Up | Y | Researcher (BG NR) | Phone; Indv | Variable over 3-month | Y | NR |
| Michiels et al, 2019 | Usual care | Pharmacist information program | Y | Pharmacist | FF; Indv | 3 sessions over 6-month | Y | Y |
| Bilger et al, 2021 | Arm 1: Usual care | Arm 2: Process; Arm 3: Outcome | Y | NR | NR | Variable over 6-month | Y | NR |
| Wong et al, 2020 | Usual care | Empowerment with cognitive training | Y | Social worker, OT, psychologist | FF; Grp | Weekly sessions over 10-week | Y | Y |
| Vos et al, 2019 | Usual care | Beyond Good Intentions | Y | Nurse | FF; Indv & Grp | 7 sessions over 12-week | Y | Y |
| Ting et al, 2021 | Usual care | Know Your Medicine | Y | Pharmacist | FF; Grp | 1 session | NR | Y |
| Du Pon et al, 2019 | Usual care | Self-Management | Y | Dietician, Nurse | FF; Grp | 2 sessions over 6-month | NR | Y |
| Miri et al, 2021 | Arm 1: CBT | Arm 2: CBT with MI | Y | Nurse | FF; Grp | Arm 1: 4 sessions over 2-week; Arm 2: 8 sessions over 3- week | NR | Y |
| Baviskar et al, 2021 | Usual care | Comprehensive diabetes management | Y | NR | FF; Grp | Bi-weekly sessions over 6-month | NR | NR |
| Rahmani et al, 2021 | Usual care | MI | Y | Therapist | FF; Grp | Weekly sessions over 8-week | NR | Y |
| Andanalusia et al, 2021 | Arm 1: Usual care | Arm 2: Education; Arm 3: Education and Pillbox | NR | Pharmacist | FF; Indv | 1 session | Y | NR |
| Rovner et al, 2020 | Arm 1: Behavioural intervention | Arm 2: Self-management education | NR | Arm 1: OT; Arm 2: CHW | FF; Indv | 8 sessions over 12-month | Y | Y |
| Selvadurai et al, 2021 | Usual care | Insulin injection re-education | NR | Pharmacist | FF; Indv | Monthly over 4-month | Y | NR |
| Kamat et al, 2021 | Usual care | KYT Adhere App | NR | NR | Mobile app | Variable over 6-month | Y | NR |
| Lim et al, 2021 | Usual care | Patients' own medicines | NR | Pharmacist | FF; Indv | 4 sessions over 12-week | Y | NR |
| Wang et al, 2021 | Usual care | Management based on family and organization | NR | Nurse | FF; Indv | Variable over 12-month | Y | NR |
| Rosli et al, 2021 | Usual care | Home medication review | NR | Pharmacist, Doctor | FF; Indv | 3 visits over 6-month | Y | Y |
| Vaughan et al, 2021 | Usual care | Medication access & education | NR | CHW | FF, phone, msg; Indv & Grp | Monthly visits & weekly call/ message over 6-month | Y | Y |
| Poonprapai et al, 2022 | Usual care | Family-based intervention | NR | Pharmacist | Mobile app | 3–5 infographics daily over 3-month | Y | NR |
| Sarayani et al, 2018 | Usual care | Education | NR | Pharmacist | Phone; Indv | 16 calls over 3-month | Y | Y |
| Withidpanyawong et al, 2019 | Usual care | Family based intervention | NR | Pharmacist | FF, phone; Indv, Grp | 4 visits & 2 calls over 9-month | Y | NR |
| VanEpps et al, 2018 | Arm 1: Usual care | Arm 2: Process; Arm 3: Outcome; Arm 4: Combined | NR | NR | NR | Variable over 6-month | Y | NR |
| Witry et al, 2019 | Arm 1: Usual care | Arm 2: Education + Medsync; Arm 3: Medsync | NR | Pharmacist | Arm 2: FF, phone; Indv, Grp; Arm 3: FF, phone; Indv | Variable over unclear duration | Y | Y |
| Asante et al, 2020 | Usual care | Mobile phone intervention | NR | Nurse | Phone; Indv | 16 calls over 12-week | Y | Y |
| Song et al, 2022 | NR | Joint pharmacy clinic | NR | Pharmacist | FF, phone; Indv | Variable over 3-month | Y | NR |
| Goruntla et al, 2019 | Usual care | Counselling and reminder | NR | Pharmacist | FF; Msg; Indv | 3 visits & daily messages over 6-month | Y | NR |
| Abubakar et al, 2021 | Usual care | Diabetes management | NR | Pharmacist | FF; Indv | 1 session | Y | NR |
| Aguiar et al, 2018 | Usual care | Collaborative care model | NR | Pharmacist | FF & phone; Indv | Variable over 12-month | Y | NR |
| Doupis et al, 2019 | Usual care | Supervised self-management | NR | Doctors, Researcher (BG NR) | FF & Phone; Indv | Variable over 8-month | Y | Y |
| Nazir et al, 2020 | Usual care | MTM | NR | Pharmacist | FF; Indv | Variable over 12-week | Y | NR |
| Munsour et al, 2020 | Usual care | Customised medicine information | NR | Pharmacist | FF; Indv | 3 sessions over 6-month | Y | Y |
| Alison et al, 2020 | Usual care | Diabetes Medication Therapy Adherence Clinic | NR | Pharmacist | FF; Indv | At least 4 visits over 9-month | Y | NR |
| Yasmin et al, 2020 | Usual care | Interactive voice call and call centre | NR | Call centre: Doctor | Phone; Indv | 1 call every 10 days over 1-year | Y | NR |
| Pinto et al, 2018 | Arm 1: PB+ Medsync | Arm 2: BP+ Medsync;  Arm 3: PB+ MTM+ Medsync;  Arm 4: BP+ MTM+ Medsync + Delivery | NR | Arm 3 & 4: Pharmacists | Arm 3 & 4: FF; Indv | Variable over 12-month | Y | Y |
| Huang et al, 2019 | Usual care | Medisafe app | NR | NR | Mobile app | Variable over 12-week | NR | Y |
| Choomai et al, 2021 | Arm 1: Education | Arm 2: Education + Insulin injection demonstration | NR | Doctor | FF; Indv | 1 session | NR | NR |
| Zheng et al, 2019 | Usual care | Education | NR | NR | FF; Grp | 1 session | NR | NR |
| Adepu et al, 2018 | Usual care | Education | NR | Pharmacist | FF; Indv | 4 sessions over 3-month | NR | NR |
| Kim et al, 2018 | Arm 1: IR BD formulation | Arm 2: SR OD formulation | NR | NR | NA | Over 24-week | NR | NR |
| Jayasree et al, 2019 | Usual care | Behaviour change communication | NR | Researcher (BG NR) | Phone; Indv | 2 times a week over 2-month | NR | NR |
| Ebid et al, 2022 | Usual care | Pharmacist intervention | NR | Pharmacist | FF; Indv | Variable over 6-month | NR | NR |
| Kandasamy et al, 2019 | NR | Patient counselling | NR | Pharmacist | FF; Indv | 4 sessions over 9-month | NR | NR |
| Machry et al, 2021 | Arm 1: Syringe | Arm 2: Pen | NR | Researcher (BG NR) | FF; Indv | Monthly over 24-week | NR | NR |
| Patel et al, 2019 | Arm 1: Basal-bolus insulin | Arm 2: Combination therapy | NR | NR | NR | Over 6-month | NR | NR |
| Chaudhary et al, 2020 | Usual care | Education and counselling | NR | Pharmacist | FF; Indv | 1 session | NR | NR |
| Wungrath et al, 2021 | Usual care | Counseling | NR | Researcher (BG NR) | Msg & phone; Indv | Variable over 7-week | NR | NR |

BG: Background; BP: Blister Pack; CHW: Community Health Worker; CBT: Cognitive behaviour therapy; FF: Face to face; Grp: Group; Indv: Individual; IR BD: Immediate release twice -daily; Medsync: Medication synchronisation; MTM: Medication Therapy Management; MI: Motivational interview; MSG: Message; NA: Non-applicable; NR: Not reported; OT: Occupational therapist; PB: Pill bottle; SR OD: Sustained release once-daily; Y: Yes

# ESM 4: Individual BCTs and success of all interventions

| Study | BCTs for each intervention | Total number of BCTs | Successful?  (Yes: Y; No: N) |
| --- | --- | --- | --- |
| Ranjbaran et al, 2022 | 1.1; 1.2; 1.4; 2.2; 3.1; 3.2; 3.3; 4.1; 6.1; 8.1; 9.2; 12.5 | 12 | Y |
| Farmer et al, 2021 | 3.1; 7.1 | 2 | N |
| Dang et al, 2020 | 1.4; 3.2; 6.1; 8.1 | 4 | N |
| Benson et al, 2019 | 1.1; 1.2; 3.1; 3.2; 9.1 | 5 | Y |
| Nelson et al, 2021 | 1.2; 2.2; 3.1 | 3 | N |
| Trevisan et al, 2020 | 1.2; 1.4; 3.1 | 3 | Y |
| Varming et al, 2019 | 1.1; 1.2; 1.5; 3.1; 3.2; 9.1 | 6 | N |
| Gautier et al, 2021 | 3.1; 4.1; 7.1; 9.1 | 4 | Y |
| Najafpour et al, 2021 | 1.2; 2.2; 3.1; 4.1; 6.1; 6.2; 8.1; 9.1 | 8 | N |
| Razmara et al, 2018 | 1.2; 2.2; 3.2; 3.3 | 4 | N |
| Michiels et al, 2019 | 3.1; 4.1; 5.1; 9.1 | 4 | N |
| Bilger et al, 2021 | Arm 2 (Process): 10.1 | 1 | Y |
|  | Arm 3 (Outcome): 10.8 | 1 | Y |
| Wong et al, 2020 | 1.1; 1.4; 3.1; 3.3; 4.1; 6.1; 8.1; 9.1 | 8 | N |
| Vos et al, 2019 | 1.1; 1.4; 1.5; 3.1; 4.1; 5.1; 6.1; 8.1; 9.1 | 9 | N |
| Ting et al, 2021 | 1.2; 2.2; 3.2; 4.1; 6.1; 8.1; 9.1 | 7 | Y |
| Du Pon et al, 2019 | 1.1; 1.4; 3.2; 4.1; 5.1; 6.1; 8.1 | 7 | Y |
| Miri et al, 2021 | Arm 1 (CBT only): 3.1; 5.1; 9.1; 11.2 | 4 | Y |
|  | Arm 2 (CBT + MI): 1.3; 3.1; 5.1; 9.1; 9.2 | 5 | Y |
| Baviskar et al, 2021 | 1.1; 1.5; 2.2; 3.1; 3.3; 4.1; 6.1; 8.1 | 8 | N |
| Rahmani et al, 2021 | 1.2; 1.4; 3.1; 4.1; 4.2; 5.2; 7.1; 8.1; 9.1; 9.2; 10.3 | 11 | Y |
| Andanalusia et al, 2021 | Arm 2 (Education): 3.1; 4.1 | 2 | Y |
|  | Arm 3 (Education + Pillbox): 3.1; 4.1; 12.5 | 3 | Y |
| Rovner et al, 2020 | Arm 1 (OT behavioural): 1.1; 1.2; 1.4; 7.1; 8.3; 9.1; 11.3; 12.1 | 8 | N |
|  | Arm 2 (CHW education): 3.1 | 1 | N |
| Selvadurai et al, 2021 | 4.1; 9.1; 12.5 | 3 | Y |
| Kamat et al, 2021 | 2.3; 3.1; 7.1; 10.1 | 4 | N |
| Lim et al, 2021 | 2.2 | 1 | Y |
| Wang et al, 2021 | 2.3; 3.2; 3.3; 9.1 | 4 | Y |
| Rosli et al, 2021 | 1.5; 2.2; 3.1; 4.1; 5.1; 9.1 | 6 | N |
| Vaughan et al, 2021 | 1.2; 3.1; 4.1; 6.1; 8.1 | 5 | Y |
| Poonprapai et al, 2022 | 3.2; 3.3; 4.1; 5.1; 5.2; 7.1; 9.1 | 7 | Y |
| Sarayani et al, 2018 | 1.2; 3.2; 9.1 | 3 | Y |
| Withidpanyawong et al, 2019 | 1.2; 3.1; 3.2; 3.3; 4.1; 6.2; 9.1 | 7 | Y |
| VanEpps et al, 2018 | Arm 2 (Process): 10.1 | 1 | N |
|  | Arm 3 (Outcome): 10.8 | 1 | N |
|  | Arm 4 (Process + Outcome): 10.1; 10.8 | 2 | N |
| Witry et al, 2019 | Arm 2 (Education + Med Sync): 1.2; 4.1; 5.1; 6.1; 8.1; 9.1; 11.3 | 7 | N |
|  | Arm 3 (Med sync): 1.2; 9.1; 11.3 | 3 | N |
| Asante et al, 2020 | 1.1; 1.4; 3.2; 9.1 | 4 | N |
| Song et al, 2022 | 1.4; 2.3; 3.2; 4.1; 9.1; 12.5 | 6 | Y |
| Goruntla et al, 2019 | 1.4; 3.1; 4.1; 5.1; 7.1; 9.1 | 6 | Y |
| Abubakar et al, 2021 | 4.1; 9.1 | 2 | Y |
| Aguiar et al, 2018 | 1.2; 3.1; 4.1; 9.1; 12.5 | 5 | Y |
| Doupis et al, 2019 | 3.2; 4.1; 9.1; 11.2 | 4 | Y |
| Nazir et al, 2020 | 1.2; 3.1; 5.1; 9.1 | 4 | Y |
| Munsour et al, 2020 | 12.5 | 1 | Y |
| Alison et al, 2020 | 1.1; 1.2; 1.4; 3.1; 9.1 | 5 | N |
| Yasmin et al, 2020 | 3.2; 7.1 | 2 | N |
| Pinto et al, 2018 | Arm 1 (PB + Med Sync): 3.2 | 1 | N |
|  | Arm 2 (BP + Med Sync): 3.2; 11.3 | 2 | Y |
|  | Arm 3 (PB + MTM + Med Sync): 1.4; 1.5; 3.1; 3.2; 9.1 | 5 | Y |
|  | Arm 4 (BP + MTM + Med Sync + Med delivery): 1.4; 1.5; 3.1; 3.2; 9.1; 11.3 | 6 | Y |
| Huang et al, 2019 | 2.3; 7.1 | 2 | Y |
| Choomai et al, 2021 | Arm 1 (Education): 5.1; 5.2; 9.1 | 3 | N |
|  | Arm 2 (Education + Actual insulin injection): 4.1; 5.1; 5.2; 6.1; 9.1 | 5 | Y |
| Zheng et al, 2019 | 4.1; 6.1; 8.1 | 3 | N |
| Adepu et al, 2018 | 3.1; 9.1 | 2 | N |
| Kim et al, 2018 | 11.3 | 1 | Y |
| Jayasree et al, 2019 | 3.1 | 1 | N |
| Ebid et al, 2022 | 3.1; 4.1; 5.1; 9.1 | 4 | Y |
| Kandasamy et al, 2019 | 3.1; 9.1 | 2 | N |
| Machry et al, 2021 | Arm 1 (Syringe): 4.1 | 1 | N |
|  | Arm 2 (Pen): 4.1; 11.3 | 2 | N |
| Patel et al, 2019 | Arm 2 Combination: 11.3 | 1 | Y |
| Chaudhary et al, 2020 | 3.1; 5.1; 9.1 | 3 | N |
| Wungrath et al, 2021 | 3.1; 3.2; 4.1 | 3 | Y |

BP: Blister Pack; CBT: Cognitive behaviour therapy; CHW: Community Health Worker; Medsync: Medication synchronisation; MTM: Medication Therapy Management; MI: Motivational interview; OT: Occupational therapist; PB: Pill bottle

# ESM 5: Proportion of individual BCTs across successful & non-successful interventions

| Individual BCTs | Number of interventions | Successful interventions | | Non-successful interventions | | Proportionally higher in successful or non-successful interventions? |
| --- | --- | --- | --- | --- | --- | --- |
|  |  | Number | Percentage | Number | Percentage |  |
| 1.1 Goal setting (behaviour) | 10 | 3 | 30.0 | 7 | 70.0 | Non-successful |
| 1.2 Problem solving | 18 | 10 | 55.6 | 8 | 44.4 | *Similar |
| 1.3 Goal setting (outcome) | 1 | 1 | 100.0 | 0 | 0.0 | Successful |
| **1.4 Action planning** | **14** | **8** | **57.1** | **6** | **42.9** | **Successful** |
| 1.5 Review behaviour goal(s) | 6 | 2 | 33.3 | 4 | 66.7 | Non-successful |
| 2.2 Feedback on behaviour | 8 | 3 | 37.5 | 5 | 62.5 | Non-successful |
| **2.3 Self-monitoring of behaviour** | **4** | **3** | **75.0** | **1** | **25.0** | **Successful** |
| 3.1. Social support (unspecified) | 33 | 17 | 51.5 | 16 | 48.5 | *Similar |
| **3.2. Social support (practical)** | **19** | **13** | **68.4** | **6** | **31.6** | **Successful** |
| **3.3. Social support (emotional)** | **7** | **4** | **57.1** | **3** | **42.9** | **Successful** |
| **4.1. Instruction on how to perform the behaviour** | **29** | **19** | **65.5** | **10** | **34.5** | **Successful** |
| 4.2. Information about Antecedents | 1 | 1 | 100.0 | 0 | 0.0 | Successful |
| **5.1. Information about health consequences** | **14** | **8** | **57.1** | **6** | **42.9** | **Successful** |
| **5.2. Salience of consequences** | **4** | **3** | **75.0** | **1** | **25.0** | **Successful** |
| 6.1. Demonstration of the behaviour | 12 | 5 | 41.7 | 7 | 58.3 | Non-successful |
| 6.2. Social comparison | 2 | 1 | 50.0 | 1 | 50.0 | Equal |
| 7.1. Prompts/cues | 9 | 5 | 55.6 | 4 | 44.4 | *Similar |
| 8.1. Behavioural practice/ rehearsal | 12 | 5 | 41.7 | 7 | 58.3 | Non-successful |
| 8.3. Habit formation | 1 | 0 | 0.0 | 1 | 100.0 | Non-successful |
| **9.1. Credible source** | **36** | **21** | **58.3** | **15** | **41.7** | **Successful** |
| **9.2. Pros and cons** | **3** | **3** | **100.0** | **0** | **0.0** | **Successful** |
| 10.1. Material incentive (behaviour) | 4 | 1 | 25.0 | 3 | 75.0 | Non-successful |
| 10.3. Non-specific reward | 1 | 1 | 100.0 | 0 | 0.0 | Successful |
| 10.8. Incentive (outcome) | 3 | 1 | 33.3 | 2 | 66.7 | Non-successful |
| 11.2. Reduce negative emotions | 2 | 2 | 100.0 | 0 | 0.0 | Successful |
| 11.3. Conserving mental resources | 8 | 4 | 50.0 | 4 | 50.0 | Equal |
| 12.1. Restructuring the physical environment | 1 | 0 | 0.0 | 1 | 100.0 | Non-successful |
| **12.5. Adding objects to the environment** | **6** | **6** | **100.0** | **0** | **0.0** | **Successful** |

*Considered “similar if the percentages were 40-50% and with a difference of about 10% between the successful and non-successful interventions

# ESM 6: Number of BCTs

| Number of BCTs | Number of interventions | Number of successful interventions | Percentage of successful interventions in each row |
| --- | --- | --- | --- |
| 12 | 1 | 1 | 100.0 |
| 11 | 1 | 1 | 100.0 |
| 9 | 1 | 0 | 0.0 |
| 8 | 4 | 0 | 0.0 |
| 7 | 5 | 4 | 80.0 |
| 6 | 5 | 3 | 60.0 |
| 5 | 7 | 6 | 85.7 |
| 4 | 11 | 6 | 54.5 |
| 3 | 10 | 5 | 50.0 |
| 2 | 9 | 3 | 33.3 |
| 1 | 13 | 7 | 53.8 |

# ESM 7: Medication adherence measure

| **Study** | **Medication adherence measure** |
| --- | --- |
| Ranjbaran et al, 2022 | Morisky Medication Adherence Scale (MMAS-8)  Health Action Process Approach (HAPA) questionnaire: Includes barriers and benefits of medication adherence |
| Farmer et al, 2021 | Proportion of patients who collected ≥ 80% medication from clinic data  Medication Adherence Rating Scale (MARS-5) |
| Dang et al, 2020 | Medication prescription refill |
| Benson et al, 2019 | 1-item questionnaire on insulin use  Morisky Medication Adherence Scale (MMAS-4) |
| Nelson et al, 2021 | Adherence to Refills and Medications Scale for Diabetes (ARMS-D)  Summary of Diabetes Self-Care Activities medications subscale (SDSCA-MS) |
| Trevisan et al, 2020 | 1-item questionnaire on taking of oral antidiabetic medication: Proportion > 80%  Instrument for the Global Evaluation of Medication Adherence: whether receiving adequate dose  6-item questionnaire on Intention to adhere to medication regime |
| Varming et al, 2019 | Summary of Diabetes Self-Care Activities medications subscale (SDSCA-MS) |
| Gautier et al, 2021 | Morisky Medication Adherence Scale (MMAS-8) |
| Najafpour et al, 2021 | Morisky Medication Adherence Scale (MMAS-8) |
| Razmara et al, 2018 | Self-care questionnaire: 1 dimension on regular use of medicine |
| Michiels et al, 2019 | Medication Possession Ratio (MPR) |
| Bilger et al, 2021 | Medication adherent days as per medication tracker |
| Wong et al, 2020 | Morisky Medication Adherence Scale (MMAS-4) |
| Vos et al, 2019 | Medication Adherence Rating Scale (MARS-5) |
| Ting et al, 2021 | Self-Efficacy for Appropriate Medication Use Scale (SEAMS) |
| Du Pon et al, 2019 | Medication Possession Ratio (MPR)  Drug holidays  Medication Adherence Rating Scale (MARS-5) |
| Miri et al, 2021 | Morisky Medication Adherence Scale (MMAS-8) |
| Baviskar et al, 2021 | Self-Care Inventory-Revised Version (SCI-R): 2 questions on medication intake |
| Rahmani et al, 2021 | Morisky Medication Adherence Scale (MMAS-8): Total score |
| Andanalusia et al, 2021 | Adherence to Refills and Medications Scale for Diabetes (ARMS-D) |
| Rovner et al, 2020 | Medication Event Monitoring System (MEMS) |
| Selvadurai et al, 2021 | Medication Compliance Questionnaire (MCQ) |
| Kamat et al, 2021 | Pill count scores  KYT Adhere app: Percentage actually consumed as opposed to expected to consume |
| Lim et al, 2021 | Pill Count: In terms of percentage |
| Wang et al, 2021 | Not specified |
| Rosli et al, 2021 | Pill Counting Adherence Ratio (PCAR) |
| Vaughan et al, 2021 | Clinician's notes on medication adherence |
| Poonprapai et al, 2022 | Pill Count: In terms of percentage |
| Sarayani et al, 2018 | Morisky Medication Adherence Scale (MMAS-8) |
| Withidpanyawong et al, 2019 | Pill Count  Morisky Medication Adherence Scale (MMAS-8) |
| VanEpps et al, 2018 | Medication prescription refill |
| Witry et al, 2019 | Proportion of days covered (PDC) |
| Asante et al, 2020 | 2 items (medication taking) in a self-management adherence questionnaire |
| Song et al, 2022 | Morisky Medication Adherence Scale (MMAS-8) |
| Goruntla et al, 2019 | Pill count  Visual analog scale (VAS) |
| Abubakar et al, 2021 | Morisky Medication Adherence Scale (MMAS-8) |
| Aguiar et al, 2018 | Morisky Medication Adherence Scale (MMAS-4) |
| Doupis et al, 2019 | Morisky Medication Adherence Scale (MMAS-4) |
| Nazir et al, 2020 | Morisky Medication Adherence Scale (MMAS-8) |
| Munsour et al, 2020 | Modified 8-item Morisky Medication Adherence Scale (MMAS-8) |
| Alison et al, 2020 | Not specified |
| Yasmin et al, 2020 | Questionnaire including Adherence to the medication advice |
| Pinto et al, 2018 | Proportion of days covered (PDC)  Pill count |
| Huang et al, 2019 | Adherence Starts with Knowledge-12 medication barrier (ASK-12) questionnaire |
| Choomai et al, 2021 | Insulin adherence - Asked about current insulin use during a visit |
| Zheng et al, 2019 | Summary of Diabetes Self-Care Activities medications subscale (SDSCA-MS) |
| Adepu et al, 2018 | Morisky Medication Adherence Scale (MMAS-8) |
| Kim et al, 2018 | Medication Event Monitoring System (MEMS) |
| Jayasree et al, 2019 | Morisky Medication Adherence Scale (MMAS-4) |
| Ebid et al, 2022 | Morisky Medication Adherence Scale (MMAS-4) |
| Kandasamy et al, 2019 | Morisky Medication Adherence Scale (MMAS-8) |
| Machry et al, 2021 | Pill count: Adherence to insulin by returning used vials |
| Patel et al, 2019 | Calculated time-adjusted average adherence rate for the entire duration of the study was ≥80% Product returned |
| Chaudhary et al, 2020 | Morisky Medication Adherence Scale (MMAS-8) |
| Wungrath et al, 2021 | Diabetes Medication Adherence Knowledge (DMAK) questionnaire  Diabetes Medication Adherence Behaviour (DMAB) questionnaire |

# ESM 8: Risk of Bias

Domain 1: Randomisation process

Domain 2: Deviations from intended interventions

Domain 3: Missing outcome data

Domain 4: Measurement of the outcome

Domain 5: Selection of the reported studies

| Low risk | + |
| --- | --- |
| Some concerns | \| |
| High risk | - |

| \| Study \| Domain 1 \| Domain 2 \| Domain 3 \| Domain 4 \| Domain 5 \| Overall \| \| --- \| --- \| --- \| --- \| --- \| --- \| --- \| \| Ranjbaran et al, 2022 \| \| \| \| \| + \| - \| + \| High risk \| \| Farmer et al, 2021 \| \| \| - \| + \| \| \| + \| High risk \| \| Dang et al, 2020 \| - \| + \| + \| + \| + \| High risk \| \| Benson et al, 2019 \| + \| - \| + \| \| \| - \| High risk \| \| Nelson et al, 2021 \| + \| + \| + \| \| \| + \| Some concerns \| \| Trevisan et al, 2020 \| + \| \| \| + \| + \| + \| Some concerns \| \| Varming et al, 2019 \| \| \| - \| + \| - \| + \| High risk \| \| Gautier et al, 2021 \| - \| \| \| + \| - \| + \| High risk \| \| Najafpour et al, 2021 \| \| \| - \| + \| + \| - \| High risk \| \| Razmara et al, 2018 \| - \| - \| - \| - \| \| \| High risk \| \| Michiels et al, 2019 \| \| \| + \| + \| + \| + \| Some concerns \| \| Bilger et al, 2021 \| + \| + \| + \| + \| \| \| Some concerns \| \| Wong et al, 2020 \| \| \| - \| - \| \| \| - \| High risk \| \| Vos et al, 2019 \| \| \| \| \| - \| \| \| + \| High risk \| \| Ting et al, 2021 \| + \| \| \| + \| + \| + \| Some concerns \| \| Du Pon et al, 2019 \| - \| \| \| + \| - \| - \| High risk \| \| Miri et al, 2021 \| + \| \| \| + \| \| \| + \| Some concerns \| \| Baviskar et al, 2021 \| + \| - \| + \| - \| - \| High risk \| \| Rahmani et al, 2021 \| \| \| \| \| + \| - \| - \| High risk \| \| Andanalusia et al, 2021 \| \| \| \| \| - \| - \| + \| High risk \| \| Rovner et al, 2020 \| + \| + \| + \| \| \| - \| High risk \| \| Selvadurai et al, 2021 \| \| \| \| \| + \| \| \| + \| Some concerns \| \| Kamat et al, 2021 \| + \| \| \| + \| \| \| - \| High risk \| \| Lim et al, 2021 \| \| \| \| \| + \| + \| + \| Some concerns \| \| Wang et al, 2021 \| \| \| \| \| + \| - \| + \| High risk \| \| Rosli et al, 2021 \| \| \| + \| + \| \| \| + \| Some concerns \| \| Vaughan et al, 2021 \| \| \| + \| + \| - \| - \| High risk \| \| Poonprapai et al, 2022 \| - \| - \| + \| + \| + \| High risk \| \| Sarayani et al, 2018 \| + \| \| \| + \| - \| + \| High risk \| \| Withidpanyawong et al, 2019 \| \| \| \| \| + \| \| \| + \| Some concerns \| \| VanEpps et al, 2018 \| \| \| + \| \| \| + \| + \| Some concerns \| \| Witry et al, 2019 \| - \| - \| + \| - \| + \| High risk \| \| Asante et al, 2020 \| + \| \| \| + \| - \| + \| High risk \| \| Song et al, 2022 \| \| \| \| \| + \| \| \| + \| Some concerns \| \| Goruntla et al, 2019 \| \| \| \| \| + \| \| \| + \| Some concerns \| \| Abubakar et al, 2021 \| \| \| \| \| + \| - \| + \| High risk \| \| Aguiar et al, 2018 \| + \| - \| + \| - \| + \| High risk \| \| Doupis et al, 2019 \| - \| - \| + \| \| \| + \| High risk \| \| Nazir et al, 2020 \| \| \| \| \| + \| - \| + \| High risk \| \| Munsour et al, 2020 \| + \| \| \| + \| \| \| + \| Some concerns \| \| Alison et al, 2020 \| + \| + \| + \| \| \| + \| Some concerns \| \| Yasmin et al, 2020 \| \| \| \| \| \| \| \| \| + \| Some concerns \| \| Pinto et al, 2018 \| \| \| \| \| + \| + \| + \| Some concerns \| \| Huang et al, 2019 \| \| \| \| \| + \| \| \| + \| Some concerns \| \| Choomai et al, 2021 \| + \| \| \| + \| - \| + \| High risk \| \| Zheng et al, 2019 \| + \| - \| + \| - \| \| \| High risk \| \| Adepu et al, 2018 \| \| \| \| \| + \| - \| + \| High risk \| \| Kim et al, 2018 \| \| \| \| \| - \| \| \| - \| High risk \| \| Jayasree et al, 2019 \| \| \| \| \| + \| \| \| + \| Some concerns \| \| Ebid et al, 2022 \| \| \| + \| + \| - \| + \| High risk \| \| Kandasamy et al, 2019 \| \| \| - \| + \| - \| - \| High risk \| \| Machry et al, 2021 \| + \| + \| + \| \| \| - \| High risk \| \| Patel et al, 2019 \| \| \| \| \| + \| \| \| + \| Some concerns \| \| Chaudhary et al, 2020 \| - \| - \| + \| - \| + \| High risk \| \| Wungrath et al, 2021 \| \| \| \| \| + \| \| \| + \| Some concerns \| \|  \| Number of studies with the overall grading \| \| \| \| Low risk \| 0 \| \|  \|  \|  \|  \| Some concerns \| 21 \| \| High risk \| 34 \| |
| --- | --- | --- | --- | --- | --- | --- | --- | --- | --- | --- | --- | --- | --- | --- | --- | --- | --- | --- | --- | --- | --- | --- | --- | --- | --- | --- | --- | --- | --- | --- | --- | --- | --- | --- | --- | --- | --- | --- | --- | --- | --- | --- | --- | --- | --- | --- | --- | --- | --- | --- | --- | --- | --- | --- | --- | --- | --- | --- | --- | --- | --- | --- | --- | --- | --- | --- | --- | --- | --- | --- | --- | --- | --- | --- | --- | --- | --- | --- | --- | --- | --- | --- | --- | --- | --- | --- | --- | --- | --- | --- | --- | --- | --- | --- | --- | --- | --- | --- | --- | --- | --- | --- | --- | --- | --- | --- | --- | --- | --- | --- | --- | --- | --- | --- | --- | --- | --- | --- | --- | --- | --- | --- | --- | --- | --- | --- | --- | --- | --- | --- | --- | --- | --- | --- | --- | --- | --- | --- | --- | --- | --- | --- | --- | --- | --- | --- | --- | --- | --- | --- | --- | --- | --- | --- | --- | --- | --- | --- | --- | --- | --- | --- | --- | --- | --- | --- | --- | --- | --- | --- | --- | --- | --- | --- | --- | --- | --- | --- | --- | --- | --- | --- | --- | --- | --- | --- | --- | --- | --- | --- | --- | --- | --- | --- | --- | --- | --- | --- | --- | --- | --- | --- | --- | --- | --- | --- | --- | --- | --- | --- | --- | --- | --- | --- | --- | --- | --- | --- | --- | --- | --- | --- | --- | --- | --- | --- | --- | --- | --- | --- | --- | --- | --- | --- | --- | --- | --- | --- | --- | --- | --- | --- | --- | --- | --- | --- | --- | --- | --- | --- | --- | --- | --- | --- | --- | --- | --- | --- | --- | --- | --- | --- | --- | --- | --- | --- | --- | --- | --- | --- | --- | --- | --- | --- | --- | --- | --- | --- | --- | --- | --- | --- | --- | --- | --- | --- | --- | --- | --- | --- | --- | --- | --- | --- | --- | --- | --- | --- | --- | --- | --- | --- | --- | --- | --- | --- | --- | --- | --- | --- | --- | --- | --- | --- | --- | --- | --- | --- | --- | --- | --- | --- | --- | --- | --- | --- | --- | --- | --- | --- | --- | --- | --- | --- | --- | --- | --- | --- | --- | --- | --- | --- | --- | --- | --- | --- | --- | --- | --- | --- | --- | --- | --- | --- | --- | --- | --- | --- | --- | --- | --- | --- | --- | --- | --- | --- | --- | --- | --- | --- | --- | --- | --- | --- | --- | --- | --- | --- | --- | --- | --- | --- | --- | --- | --- | --- | --- | --- | --- | --- | --- | --- | --- | --- | --- | --- | --- | --- | --- | --- | --- | --- | --- | --- | --- | --- | --- |

# ESM 9: Mapping of the common BCTs in successful interventions from BCTTv1 to BCTO

| Common BCT found in successful intervention as per BCTTv1 | Closest BCT in the BCTO |
| --- | --- |
| “Credible source” (BCT 9.1) | Present information from credible influence BCT |
| “Instruction on how to perform the behaviour” (BCT 4.1) | 2 BCTs:   - Instruct how to perform behaviour BCT - Agree on how to perform behaviour BCT |
| “Social support (practical)” (BCT 3.2) | 3 BCTs:   - Advise to seek instrumental support BCT - Arrange instrumental support BCT - Deliver instrumental support BCT |
| “Action planning” (BCT 1.4) | Action planning BCT |
| “Information about health consequences” (BCT 5.1) | Inform about health consequences BCT |

Mapping was done on 11^th^ Dec 2023
